# Spotlight on mentoring undergraduate medical students using evidence-based medicine

**DOI:** 10.1186/s12909-026-09490-3

**Published:** 2026-05-25

**Authors:** Clovis Foguem, Patrick Manckoundia

**Affiliations:** 1 Auban Moët Hospital - GHU de Champagne, 137, rue de l’Hôpital Auban-Moët, BP 137, Epernay, 51205 France; 2 ULR METRICS - Université de Lille, 1 Place de Verdun, Lille, 59045 France; 3 Université Bourgogne Europe, CHU Dijon Bourgogne, Service de Médecine Interne Gériatrie, Dijon, 21000 France; 4 Université Bourgogne Europe, INSERM U1093, Cognition Action Plasticité Sensori-Motrice, Dijon, 21000 France

**Keywords:** Education, Evidence-based medicine, EBM, Medical students’ rotation, Effectiveness, Medical curriculum

## Abstract

**Introduction:**

Evidence-based medicine (EBM) is currently used for patient management and to draw up recommendations. However, the effectiveness of EBM-related teaching during the mentoring of undergraduate medical students has not been widely evaluated. In this work, we aimed to (1) review published articles on EBM-related teaching during undergraduate clinical rotations and (2) to investigate the effectiveness of the EBM mentoring strategies used.

**Methods:**

We performed a structured narrative review of articles or abstracts published in English up to January, 2026 focused on the application of EBM during undergraduate clinical rotations, and indexed in *Pubmed*,* Sciencedirect and Scopus* databases. Publications were screened using a combination of four ‘key terms’: *evidence-based medicine*, *EBM*, *undergraduate medical students* and *clinical rotation.*

**Results:**

Almost all retrieved publications reported positive outcomes following EBM approaches or courses implemented during clinical rotations.

**Discussion:**

We identified several factors related to EBM mentoring that can influence the learning process of medical students (knowledge, skills, attitudes…) during clinical rotations. Including EBM in the undergraduate curriculum improved evidence-based practices and was found to be highly effective. Only 19 of the 188,859 initially retrieved publications met the inclusion criteria and were incorporated in this review.

**Conclusion:**

EBM improves instruction and tutoring during undergraduate clinical rotations; it strengthens critical thinking and often opens up additional perspectives, enabling progress. EBM should be included in undergraduate medical curricula and rotations, worldwide.

**Supplementary Information:**

The online version contains supplementary material available at 10.1186/s12909-026-09490-3.

## Introduction

First described in 1990 by Guyatt [1], evidence-based medicine (EBM) integrates and encourages the implementation of the best available published clinical evidence along with patients’ values or choices and the caregiver’s medical expertise, to optimize informed medical decisions. EBM aims to improve patient health by implementing the best available evidence to decisions affecting health outcomes. EBM can also be used to enhance mentoring, a simulation-based education approach that aims to help medical students develop into competent, well-rounded physicians [2], and could or should be considered one of the keys to a successful and fulfilling medical career. More specifically, mentoring is an informal process allowing the transmission of knowledge, experience, wisdom, social capital, or psychosocial support from mentors or relatives who possess the above characteristics, to mentees (dedicated students) for their professional development during a coaching or working session, such as rotations for medical students.

Effective EBM training is also reported to enhance the knowledge and skills of healthcare professionals [3]. However, rigorous studies of EBM approaches in medical education are rare [4, 5]. This is especially true in healthcare contexts dealing with populations at the extremes of life, where metabolism and physiology are slightly different (e.g. very young infants or elderly people), due to the adaptability of its implementation in these population groups, or dealing with acute situations (e.g. emergency or intensive care). Also, there is insufficient data about EBM education in undergraduate medical mentoring programs in most countries [6].

The perpetuation of good clinical and therapeutic practices among doctors should certainly involve teaching EBM to medical students [7]. From this perspective, numerous initiatives have sprung up to promote EBM, including lectures, traditional conferences, journal clubs, seminars, workshops, e-learning classes, webinars, competitions and educational games [8].

The main aim of this topical and structured narrative literature review involving publications in English and indexed in the *PubMed*,* ScienceDirect and Scopus* databases was to assess the effectiveness of teaching and learning EBM in undergraduate medical curricula worldwide, including indidactic lectures, clinical teaching, conferences, laboratories, student advising or student preceptorships.

## Methods

In order to fulfill the aims of this paper, we searched for abstracts, accepted preprints, academic dissertations or articles published in English, with a view to only considering publications with an international impact. The authors searched multiple sources including medical journals, educational journals, and electronic databases indexed in three of the largest bibliographic search engines for medicine and biology: PubMed, ScienceDirect, and Scopus databases. They were not assisted by a health librarian or information specialist. Grey literature was not included.

**Fig. 1:**
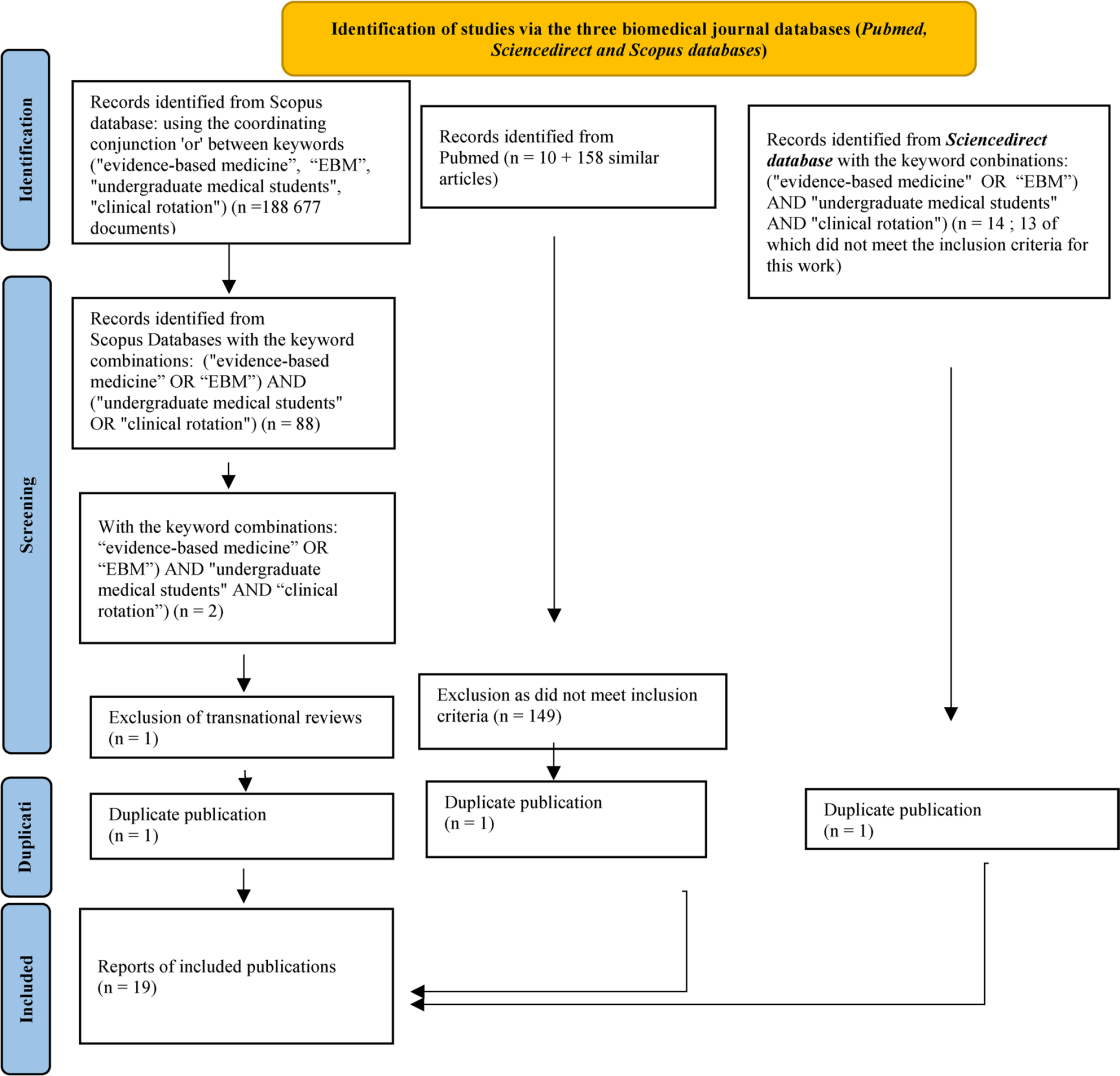
Flow diagram of the article search and selection processes (PRISMA figure) [9]

The framework guiding this structured narrative review is supported by PRISMA-informed narrative synthesis combined with a bibliometric analysis. First of all, the bibliometric analysis was done using Biblioshiny based on four keywords (“evidence-based medicine”, “EBM”, “undergraduate medical students” and “clinical rotation”). The combination of these keywords using Boolean operators was as follows: (“Evidence-based medicine” OR “EBM”) AND “undergraduate medical students” AND “clinical rotation”. Therefore, based on the results of the bibliometric analysis, a second detailed analysis of the retrieved publications was done using PRISMA-informed narrative synthesis [9] as shown in Fig. 1 and in the Appendix. Metadata of selected publications were collected up to January 2026. Duplicated publications and homonyms were excluded (Fig. 1). Then, the inclusion criteria for the selected articles were the presence of at least two study groups and of at least one comparative analysis. Transnational reviews were excluded due to the increased risk of biases, such as selection bias depending on the country, inadequate or lack of blinding between countries, and attrition bias [10].

Thirdly, in order to ensure the highest possible relevance, both authors participated in reviewing the pre-selected publications. Ultimately, only publications retained by both co-authors were included in the review.

The following data were extracted, when possible, from the retrieved trials: country and setting, year of publication, main aims of the study, mentoring methods or models, number of medical students enrolled in the trial, length of follow-up, type of intervention or research design, EBM program evaluation, program outcomes or effectiveness (including improvement of clinical skills or satisfaction with clinical skills, and statistical significance where applicable).

We analyzed the implementation of EBM during clinical rotations and the effectiveness of the approach, as described in the studies. Significance was set at a p-value < 0.05 for statistical analyses.

The current study did not require approval from an ethics committee.

## Results

Among the 188,859 publications retrieved (188,677 from Scopus, 168 (10 and 158 related articles) from Pubmed and 14 from ScienceDirect), 24 articles were initially selected. There was one disagreement between co-authors; this article did not fully meet the inclusion criteria and was not retained. Consequently, the inter-rater reliability of Cohen’s Kappa coefficient (for inter-rater agreement) in this study is 0.92 (near perfect agreement (0.81–0.99)). Ultimately, 19 publications met the selection criteria and were included in this review (Table 1).

These 19 remaining articles were comparative studies or randomized controlled trials and were conducted in various continents, except for Africa or Latin America. Seven (7) studies were conducted in the USA, three (3) in Europe (France, Ireland and United Kingdom), five (5) in the Middle East (Jordan, Iran (2), Turkey (2)) and four in Asia (Taiwan, Thailand (2), Malaysia). Condensed summaries of these 19 articles are provided in Table 1 [11–29]. This selection of articles should be considered representative but not exhaustive considering that other articles focused on EBM were not selected because they did not meet the inclusion criteria.

We performed a descriptive analysis of the retrieved studies, and findings from these studies are presented as a narrative synthesis (Table 1). Two retrieved articles were qualitative studies. Among the 17 remaining articles, 88.23% (15/17) reported that students saw improvements after their exposure to EBM. However, in the two remaining articles (11.77%), one reported no obvious difference between EBM-exposed and non-exposed groups and the other publication found a significant decrease in comfort ratings for students using EBM to motivate patients or to manage a conflict (Table 1).

Improvements were seen in particular for undergraduate clinical skills, including gains in knowledge and improved attitudes about patients, including in some specific contexts. The main outcomes and the ‘p’ probabilities of statistically significant differences are shown in Table 1, when available.

**Table 1:** Characteristics of 19 publications on Evidence-based medicine (EBM) applications during undergraduate clinical rotations, included in the study

Study	Site	Title	Purpose	Methods	Participants	Main results
1_Holloway R et al.2004 [11]	USA	Teaching and evaluating … evidence-based medicine.	Evaluate EBM practice through an EBM teaching program for the first 2 years of medical studies.	- Implementation of a curriculum including 25 to 30 h of EBM teaching for 100 medical students (first and second years). − 5 main practical components of EBM were evaluated: formulation of well-constructed questions, search for evidence, critical appraisal, application of evidence and self-evaluation.	100 students admitted to the first year of medical school; covering the first and second years of their studies.	very early stages to determine ‘when’ and ‘how’ EBM education should be delivered during medical training due to the weak correlations found when assessing the areas of scientific knowledge (*r* = 0.23, *p* = 0.05), work habits (*r* = 0.24, *p* = 0.02), and reasoning skills (*r* = 0.31, *p* = 0.002) needed to practice EBM - Scores lacked test-retest reliability and did not correlate with components of the comprehensive assessment - Students noticed that EBM was emphasized too much, and ranked the ‘EBM test’ module as the lowest-scoring component of the comprehensive evaluation.
2_Sullivan C et al. 2022 [12]	Ireland	Preparing for Pediatrics: their Clinical Placement.	Exploring the impact of learning experiences on the development of pediatric communication skills in medical students and their preparation for clinical clerkship, as well as the impact of workshops on children’s health literacy.	New course involving experiential learning, including video-recorded consultations with simulated parents, team scenarios with a pediatric mannequin, and interactions with healthy children as part of a preschool visit, was designed and implemented; medical students led health workshops for elementary school children. Medical students’ perspectives were studied using a pre- and post-intervention questionnaire and post-intervention group discussions.	144 fourth-year medical students during their pediatric internship [All 144 (100%) of consenting students completed pre-intervention questionnaire, and only 59/144 (40.1%) of consenting students completed the post-intervention questionnaire].	- significant decrease was observed in the way students rated their comfort in using EBM when motivating patients (*p* = 0.003). and for questions relating to managing a conflict situation involving family members, - no change for the question concerning effectiveness at conducting a psychosocial assessment with an adolescent -Four themes related to how students have experienced the intervention were highlighted from eight group discussions (*n* = 35 students): Shaping student learning; Supporting student learning; Developing new skills and Feeling better prepared.
3 _Taheri H. et al. 2008 [13]	Iran	EBM for undergraduate medical students.	Determine if an EBM workshop would enhance (1) medical students’ ability to formulate clinical questions, (2) search databases and (3) their attitude towards EBM.	In this prospective quasi-experimental study, students learned and practiced how to: (1) formulate clinical questions, (2) conduct literature searches and (3) perform critical appraisals. Pre- and post-workshop assessments of the students’ ability to grasp the above points were carried out. Students’ attitudes were also assessed with Likert scale 15-item questionnaire.	24 Fifth- and sixth-year students of Isfahan’s Faculty of Medicine (15 (63%) were female).	- After the EBM workshop, medical students improved their ability to formulate clinical questions and conduct appropriate literature searches (*p* < 0.01); -Their attitudes towards learning and using EBM were also improved.
4_Foguem C and Manckoundia P. 2018 [14]	France	Experience of medical students mentoring… evidence-based medicine	Evaluate the impact of EBM on students’ practical training during hospital internships in an Acute geriatric unit.	Comparison, in retrospect, of students’ group performance at the end-of-course assessment: those who had short, structured courses in EBM-integrated semiology during the rotation and those who had not (controls).	64 undergraduate medical students (MS; 40 s and 24 third-year’s MS)	-EBM group had significantly better final evaluation results than the control group (*p* < 0.01). -Students in the EBM group were more satisfied with the hospital internship and mentoring.
5_ Cayley WE Jr. 2005 [15]	USA.	Evidence-based medicine…primary care rotation.	Introduce third-year students to the practice of EBM during primary care hospital internships and promote the worth of an evidence-based approach.	Eight items of a 27-question questionnaire were used to measure the impact on the students’ own understanding and use of EBM, during four hospital rotations including series of six-hour seminars.	27 students in their third year of medicine	Significant improvement of self-reported understanding in EBM and greater ease in critical evaluation after analysis of the questionnaires given to students, before and after completion of the study program (p <. 0.05).
6 _ Cheng HM et al. 2012 [16]	Taiwan	Two strategies to intensify …randomised controlled trial.	Appreciate the effects of the EBP curriculum through two integrated clinical teaching strategies: (1) clarify EBP principles through didactic discourses or (2) reflect on the problems of patient care experiences with an EBP approach through case conferences.	A randomized controlled trial with before (at baseline)-and-after (2 weeks later) evaluations to examine the effects of EBP during a General Medicine’ rotation. All included students were evaluated on their knowledge (EBP-K), attitude (EBP-A), personal application (EBP-P) and anticipated future use (EBP-F) of EBP.	94 final-year medical students (7-year course of study with 2 years of pre-medical training) during hospital internships in a General medicine department.	After the two weeks of EBM instruction, all scores were improved in both groups. However, students in Group A had significantly higher post-intervention scores than those in Group B for EBP-K (*p* < 0.01) and EBP-P (*p* < 0.001). In contrast, EBP-A and EBP-F scores were not statistically different between the two groups.
7_West CP, McDonald FS. 2008 [17]	USA.	Evaluation of a longitudinal…: a pilot study.	Evaluate a longitudinal EBM-based medical education program using a validated tool.	Medical students’ EBM attitudes and knowledge were assessed. The first part of the training consisted of a “short course” with didactic and small group sessions, at the end of the second year. The second part integrated work on evidence-based medicine during third-year clinical rotation. 15-item Berlin Questionnaire was realized before the course, after the short course and at the end of the third year.	32 medical students aspiring to begin third-year clinical rotations immediately start of the survey at the end of the second year of medical school.	EBM knowledge scores heightened by 2.8 points at the end of the second year (*p* = 0.0001) and by 3.7 points at the end of the third curriculum year (*p* < 0.0001). Self-rated EBM knowledge marked up by 0.8 and 1.1 points from baseline (*p* = 0.0006 and *p* < 00001, respectively). EBM was rated as very important for medical education and clinical practice at all time points, with a peak after the short course.
8_Sastre EA, et al. 2011 [18]	USA	Teaching evidence-based medicine: clinical documentation.	Evaluate the impact of ‘*Teaching literature search techniques to third-year medical students through a brief workshop*’ on students’ use of EBM-related elements during their clinical placements, and on the quality of EBM integration into clinico-biological observations of hospitalized patients. .	A workshop was set up to introduce EBM-related elements to Internal Medicine interns. Pre- and post-workshop evaluation included students’ attitudes towards EBM, citations of EBM resources in their clinical observations, and the quality of the EBM component in the observation discussion. Analysis of the computer logs of the devices used by the students (computers and telephones) made it possible to assess the students’ online research attempts.	115 Third-year medical students during internships in Internal medicine	Workshop significantly improved student confidence in EBM (*p* < 0.001), increased use of EBM resources and improved integration of EBM into inpatient notes. After this exposure, students were more comfortable using EBM (*p* < 0.001), and made greater use of EBM resources (*p* < 0.031)
9_Barghouti FF et al. 2013 [19]	Jordan	Short course in evidence-based medicine improves… medical students: a before-and-after study.	Evaluate the effectiveness of a short course on EBM for improving the knowledge and skills of undergraduate medical students during rotation through the Department of Family Medicine at Jordan University Hospital, and indicate the possibility of integrating EBM into their curriculum.	A two-week short course including lectures, seminars, online research and worksheets was delivered to the 54 fifth-year medical students included in the study, from September 1 to mid-December 2011. Pre- and post-course evaluations, seminars and research relating to EBM were carried out.	54 fifth-year medical students on internships in the department of Family medicine	Participants obtained a mean score of 26.7 out of 200 on the pre-test and 119.5 on the post-test. This resulted in a largely statistically significant difference in means between pre-test and post-test (confidence interval of [84.7, 101.0]; (*p* < 0. 0001).
10_Hadvani T et al. 2021 [20]	USA	Effectiveness of Modalities to Teach Evidence Based Medicine to Pediatric Clerkship Students: A Randomized Controlled Trial	To evaluate the effectiveness of a TDS versus a SPM on the application of EBM skills in medical students during their pediatric clerkship.	A randomized controlled trial from June 2017 to June 2018: students randomized into TDS or SPM module during each two-week block. All students completed a CAT of evidence related to a clinical question in a standardized assessment form and self-reflected on the EBM process.	127 students on clinical pediatric rotation in a quaternary care children’s hospital .	Overall, there was no significant difference in mean CAT scores for the TDS groups versus the SPM groups (*p* = 0.65). There was no significant difference between the SPM and TDS groups for knowledge (*p* = 0.66), attitudes (*p* = 0.97), confidence (*p* = 0.55) and access to evidence (*p* = 0.27). Both groups showed significant gains in knowledge, attitudes, confidence, and accessing evidence from baseline to post-course. Overall, SPM learning module is as effective as a TDS module in applying EBM concepts and knowledge to patient care.
11_ Çakmakkaya ÖS. 2021 [21]	Turkey	Formal evidence-based medicine instruction in Turkish undergraduate medical education: an initial evaluation.	Evaluation the effect of a formal EBM instruction to the curriculum on students’ knowledge and skills by using the ‘Fresno’ Turkish adapted Test.	An experimental investigation using pre- and post-test evaluations, circumscribing ‘a five-week EBM course according to Kern’s six-step curriculum development approach	78 students from the third (*n* = 30), fourth (*n* = 19) and fifth (*n* = 29) year of Cerrahpaşa Medical Faculty	-Improvement of students’ mean pre-test Fresno Test score from 49.9 ± 18.2 to 118.9 ± 26.3 post-training. -Overall students’ satisfaction score was 8.66 ± 1.09 on a 1 to 10 scale.
12_ Kumaravel B, et al. 2020 [22]	United Kingdom (UK)	A prospective study evaluating the integration of a multifaceted evidence-based medicine curriculum into early years in an undergraduate medical school.	To test the feasibility of integrating a multifaceted EBM curriculum in the early years of an undergraduate medical school, using the validated Fresno test and students’ self-reported knowledge and attitudes as they progressed through the curriculum.	The 212-point Fresno test was administered to the First-year medical students along with a locally developed questionnaire at baseline before EBM teaching in year one and at the end of EBM teaching in year two (2nd years of undergraduate medical school)	Thirty-one students participated at baseline ((1^rst^ years of undergraduate medical school) and 55 students participated at the end of second year EBM teaching	.For the 18 students who completed the Fresno at both time points, the average score increased by 38.7 marks (*p* < 0.001) after EBM teaching. Students felt confident in formulating clinical questions and in critically appraising journal articles after EBM teaching. EBM They also perceived EBM to be important to their developing practice as a doctor and for improving patient outcomes at both time points.
13_ Liabsuetrakul T, et al. 2009 [23]	Thailand	Longitudinal analysis of integrating evidence-based medicine into a medical student curriculum.	To determine changes in self-reported attitudes and skills after integration of EBM into a medical school curriculum	A pre- and post- EBM intervention study was conducted to Undergraduate medical students during 2005–2007. Fourth-year medical students were instructed in EBM by a team promoting EBM and then practiced EBM under supervision of faculty advisors. Changes in attitude and skills before studying EBM (T0) and at two points (T1 and T2) after learning about EBM were assessed.	All 259 students enrolled in the medical school over the 2-year study participated in the study; among them, 132 medical students began the course in 2005.	The results showed significantly higher scores for attitude and skills over time (P0.85). Indeed, the students’ attitudes and skills at T1 and T2 were improved significantly compared to ratings at T0 (before integration of EBM into the curriculum).
14_ Ghojazadeh M et al. 2014 [24]	Iran	Medical students’ attitudes on and experiences with evidence-based medicine: a qualitative study.	Determine the attitudes towards and experiences of medical students on evidence-based medicine (EBM).	Medical students’ attitudes about and experiences with evidence-based medicine were determined through semi-structured interviews. The context of interviews was analysed using the content analysis method.	Forty senior medical students (final year of undergraduate medical study) .	Medical students included globally think that: access to information, teamwork and faculty members or mentors were necessary for EBM. Students reported having used EBM for problem solving, thinking and self-confidence. On the other hand, lack of equipment and facilities, human factors and organizational factors were considered the main barriers to EBM use.
15_ Srinivasan M, et al. 2002 [25]	USA.	Early introduction of an evidence-based medicine course to preclinical medical students.	Evaluation the feasibility of introducing a 1-month problem-based EBM course using an observational research design.	Authors assessed program performance through the use of a web-based curricular component and practice exam, final examination scores, student satisfaction surveys, and a faculty questionnaire.	139 first-year medical students at a large university center.	Students demonstrated active involvement in learning EBM and ability to use EBM principles. They performed well and compared favorably with residents whom they had supervised in the past year. Students were satisfied with the EBM course. The early introduction of EBM principles as a short course to preclinical medical students is feasible and practical.
16_ Elçin M, et al. 2014 [26]	Turkey	Development and evaluation of the evidence-based medicine program in surgery: a spiral approach.	Evaluate the EBM program implemented at Hacettepe University School of Medicine (HUFM).	A spiral program for the teaching and practice of EBM was developed for the first 3 years of medical school. Following this program, a practice of EBM was included in the fourth year during the surgery clerkship, after an introductory lecture. The students worked within collaborative teams of 3–5 and practiced EBM with actual cases seen in the surgical service in which they were involved. Each student was asked to complete a questionnaire that evaluated the more theoretical program from the first 3 years and the practical application in the fourth year.	Ninety-six (96)’s fourth year medical students who attended the clinical EBM program and attended the general surgery clerkship at HUFM	Nearly half of the students stated that the preclinical years of the EBM program were ‘adequate’, but only 30% of the students indicated that the program was practical. More than 75% of the students declared that the practice of EBM in the fourth year was useful and appropriate for team-based learning.
17_ Aronoff SC, et al. 2010 [27]	USA	Integrating evidence-based medicine into undergraduate medical education: combining online instruction with clinical clerkships.	Determine if an online course in evidence-based medicine run concurrently with the clinical clerkships in the 3rd year of undergraduate medical education provided effective instruction in evidence-based medicine (EBM).	During the first 18 weeks of the 3rd year, students completed 6 online, didactic modules. Over the next 24 weeks, students developed questions independently from patients seen during clerkships and then retrieved and appraised relevant evidence. Online, faculty mentors reviewed student assignments submitted throughout the course to monitor progress. Mastery of the skills of EBM was assessed prior to and at the conclusion of the course using the Fresno test of competency.	139 3rd year medical students.	Post-course test scores (M = 77.7; 95% CI = 59-96.4) were significantly higher than pre-course scores (M = 66.6; 95% CI = 46.5–86.7), *p*< 0.001. Paired evaluations demonstrated an average improvement of 11.1 +/- 20.0 points. All of the students submitted 4 independently derived questions and successfully retrieved and appraised evidence.
18_ Liabsuetrakul T, et al. 2017 [28]	Thailand	Implementation of evidence-based medicine in a health promotion (HP) teaching block for Thai medical students.	Assess the perceived usefulness of EBM in the teaching of HP among medical students and faculty members.	A comparative study was conducted between two groups of fourth-year medical students. A one-week EBM course was conducted with half the students in the first week of the promotion teaching’ block and the other half of the students in the last week of the block. Except for the different perods of the one-week of EBM teaching, all activities in the HP block were similar. The effect on knowledge, ability and perceived application of EBM in future practice was assessed by student self-evaluations before versus after taking the EBM course, and by faculty member evaluation of the students’ end-of-block presentations. All evaluation items were rated from 1 (lowest) to 5 (highest).	135 fourth-year medical students, divided into two groups: 68 in the first group and 67 in the second.	The students’ self-evaluations of knowledge and ability on EBM between the two groups were similar. The perception that teaching EBM is beneficial in health promotion and future practice increased significantly (*p* < 0.001) in both groups. Faculty members rated higher scores for the first group than the second group, although the rating differences were not at the level of significance. 90% of the students believed that EBM was a useful addition to the teaching of HP.
19_ Lai NM, Nalliah S. 2010 [29]	Malaysia	Information-seeking practices of senior medical students: the impact of an evidence-based medicine training programme.	Assessing whether EBM training in the final six months of medical training changes our students’ information-seeking practices and their confidence in understanding and appraising clinical evidence.	Between September 2005 and February 2006, self-administered questionnaires were distributed to 65 senior medical students at the beginning and again at the end of their clerkship training during which there was a clinically-integrated EBM curriculum. The questionnaires covered the topics of their preferred sources of clinical information, online search frequencies, estimated time to retrieve an abstract, and their understanding and confidence in their critical appraisal skills.	65 senior medical students.	There were significant increases in search activities following the curriculum, for example, students who searched PubMed or Medline for more than three times per week increased from 9.7% to 31.7% (*p* < 0.001). Despite significant improvements in students’ reported understanding of journals and their confidence in critical appraisal (*p* < 0.001), there was no improvement in reported search speed, with 48.4% in the initial survey and 49.2% in the follow-up survey reporting to take 30 min or less to trace an abstract of interest (*p* = 0.979).

*Abbreviations and Acronyms:* *ACE* Assessing Competency in EBM, *BL* Blended learning, *CAT* Critical Appraisal Tool, *EBM* Evidence-based medicine, *EBP* EBM Practical Use, *DL* Didactic learning, *HP* Health promotion, *MS* Medical students, *RCT* Randomised controlled trial, *SPM* Self-paced interactive multimedia, *TDS* Traditional didactic session, *USA* United States of America

## Discussion

Although EBM is a well-known concept, its application, role and significance in undergraduate medical curricula is currently not well defined, and there is no consensus regarding how it should be delivered to undergraduate medical students. However, the majority of the quantitative publications included in this work (88.23%) highlighted the positive outcomes obtained with EBM during undergraduate medical student rotations (Table 1); the objectives and parameters evaluated in these 15 publications were factual, measurable or quantifiable. Some of the best ways to implement, and evaluate EBM-related mentoring as opposed to traditional university lectures for undergraduate medical students as an effective intervention during rotations, have been identified in this structured narrative review. Integrating EBM into hospital internships would help medical students to acquire or develop the professional skills and competencies they need for current practice and self-learning [4]. EBM processes involve the following steps: (1) formulating a clear and precise clinical question relating to the objectified clinical situation; (2) searching the literature for relevant scientific publications focused on the issue; (3) critically assessing the validity and relevance of the results found; and (4) integrating these results into patient management [11].

Some of the studies described innovative educational techniques, for example clinical vignettes (including medical history, physical examination, investigations, treatment), based on real cases after clinical examination of patients or clinical instruction at the bedside, preceded by tutorial videos [14], or clinical vignette-based interactive discussion sessions [30]. They also included reminders of semiology classes [14], simulated patient case videos [31] and related questions and responses followed by discussion referring to the latest articles on the subject [32]. Additionally, they described consensus-building small-group discussions with close faculty (mentor)-student interactions, and moderation by faculty members with timely feedback [14, 33], and theoretical learning based on practice or students’ personalized literature searches [14, 33]. Some authors suggested the need for a total training time of at least 8 h, including individual sessions lasting more than 90 min [34, 35]. This multi-dimensional approach to EBM appears to be both particularly stimulating and efficient as an educational and mentoring method for medical students.

These different elements provide students with extensive, relevant, hands-on clinical experience combining clinical and scientific fundamentals, under supervision.Universities could, for example, conceptualize and adapt these techniques to their pedagogical and cultural environments, leading to a generalization if future implementation experiences are successful.

According to the retrieved studies, the elements that appear to best support the learning and assimilation of EBM by medical students are: (1) the acquisition of global knowledge and clinical skills in undergraduate programs; (2) the assessment of autonomy during clinical patient examinations or clinical case presentations at the end of the rotations; (3) student satisfaction; (4) EBM teaching methods and assessment in clinical practice; and (5) assessment of the available literature based on essays and surveys. Student-related characteristics, such as respectfulness, punctuality, enthusiasm, proactivity, confidence, knowledge, and the ability to prioritize and to respond to criticism, also influenced the effectiveness of the mentoring relationship. This mentoring relationship is important for the establishment of feedback loops, improving student-tutor relationships and resulting in highly effective mentoring [36]. It can be further strengthened when medical students take a proactive approach. EBM also improved self reported attitude and skills during and after rotations [23, 25]. Furthermore, earlier exposure to EBM should enable students to acquire the skills needed to evaluate the relevance and quality of scientific articles as well as their limitations and their outcomes [33]. EBM could serve as a link between learning (theoretical and practical) and clinical decision making [16].

In parallel, some features of effective educational EBM programs include the mentor’s qualities and attitudes: availability, integrity, impartiality, listening and good communication skills, positive attitude and encourage student empowerment, inter alia. In addition, self-implemented EBM mentoring and an emphasis on bedside medical education enhances the clinical skills and knowledge necessary to acquire more autonomy when conducting clinical examinations [5, 34]. Mentors should not only teach students what to do directly, but also what they should do to acquire new clinical skills or theoretical knowledge. It has also been shown that the use of multiple teaching schemes in EBM gives better results [14].

Effective EBM mentorships also help students develop a body of knowledge about EBM that can benefit both teachers and medical students [37] by improving general medical culture, practice and expertise.

It is assumed that medical students who are independent and creative thinkers are more likely to be better doctors. These creative students appear to play a more active role in the discovery process and in their own learning; they also have higher research productivity [38].

Undergraduate rotations are an essential part of medical education. They provide the student’s first experiences in patient care in the hospital environment. Young undergraduate medical students who are in the process of assimilating medical theory are generally keen to discover and to experience clinical practice – clinical rotations provide this opportunity and may be the ideal time to initiate EBM. Many proponents of EBM prioritize hierarchies of evidence from various sources in order to evaluate them [39] and to teach medical students. However, it is often difficult to apply evidence prioritization in certain circumstances, such as in the extremes of age or in acute or crisis situations [40].

To be fully effective, EBM should not be restricted to randomized trials and meta-analyses. Indeed, without clinical expertise, clinical practice can be dictated by scientific evidence and trials; and even excellent scientific evidence may be inapplicable or inappropriate for a given patient. Likewise, in the absence of proven, validated and up-to-date data, medical practices can quickly become obsolete or detrimental to patients. EBM is therefore an optimal combination of clinical expertise, the best available research data and the unique characteristics of a patient, all of which guide and ensure optimal patient care [1].

Notwithstanding that the effectiveness EBM has been criticized in some circumstances, as stated above [40], it contributes to better medical reasoning and more structured approaches and should thus be included in the clinical training of undergraduate medical students [41]. Some critics have suggested that EBM is a dangerous innovation initiated by administrative authorities to reduce health care costs or to limit hospital autonomy. In some studies, the application of EBM in medical units (evidence-based practice) did indeed lead to a significant reduction in unnecessary medical procedures and healthcare costs in the outpatient setting, although this had no impact on patient satisfaction [42]. Overall, cost-effectiveness data are lacking in most studies, despite the potential beneficial effects on career choices, performance improvements, and higher research productivity.

In a context of mentoring, medical professionals take the role of mentors on a volunteer, not-for-profit basis. There are others limitations to the implementation or expansion of EBM during clinical rotations, including large student groups (more than eight or ten students per group during the rotation), or the short rotation periods [13]. In addition, the difficulty in demonstrating the effectiveness of EBM learning and mentoring is often related to study protocols, which may not include control groups, validated assessment tools, or integrated strategies for undergraduates [16].

Two of the 19 articles included in this analysis (10.52% of selected publications) did not report positive outcomes after the implementation of EBM. This was the case for instance, in the work of Holloway et al. who evaluated the effects of EBM in first-and second-year medical students [11]. This raises questions about how and when EBM should be introduced into the medical curriculum to ensure optimal effects. In the light of the results obtained by Holloway et al. [11], it might be more appropriate to introduce EBM from the third year of medical school onwards, after students have assimilated some of the scientific and medical fundamentals.

The second study that did not observe positive effects reported that medical students found it difficult to use EBM to manage conflictual situations or to motivate patients [19]. This may be due to the emotional or affective components that are often present during a crisis or conflict situation, factors that are not fully considered in EBM.

Finally, the fact that EBM can be time-consuming to implement during hospital internships could also be an obstacle to its expansion or large-scale use.

### Strengths and limitations

Our literature review may have some limitations. First, we could have used additional scientific search engines and databases. However, we believe that this bias is minor considering our use of three large databases (PubMed, ScienceDirect and Scopus) referencing a vast number of articles in science, medicine and pedagogy and with a large bibliographic scope. Another limitation is that only articles published in English were included, but our intention was to focus on articles with international resonance. The main limitation of this paper is the small number of included articles since, of the 188,859 initially preselected articles only 19 were retained after a more thorough assessment of their contents. Moreover, the lack of health librarian or information specialist involved in the search strategy development, given the complexity of the search and volume of records retrieved, is also another limitation of this study. Our work also has limitations inherent to its retrospective nature. Additional quality assessments were not done for the included articles considering the already strict selection criteria.

Moreover, EBM can be criticized for its tendency to place too much reliance on clinical studies and systematic reviews. One of the strengths of this study is our attempt to counter this tendency by highlighting work that combines clinical examinations and bedside assessments with reasoning mechanisms that include the results of randomized studies and systematic reviews.

### Implications for practice

Most of the data found in this literature review present a positive view of EBM in medical education. We suggest that the implementation of EBM could lead to higher standards for education, particularly for medical students, and contribute to the training of well-rounded doctors. However, EBM can be ineffective or inefficient when it comes to assessing parameters that cannot be quantified metrically, or when managing situations with complex components, including intense emotions.

## Conclusion

This literature review identified the advantages of EBM-based teaching during undergraduate rotations while highlighting the few limitations of this technique. Our observations suggest that, to capitalize on these positive outcomes, medical schools should include EBM in teaching curricula. However, further country-specific studies are warranted be to provide objective data that can be used by decision-makers to support the systematic implementation of EBM in teaching.

## Supplementary Information

Supplementary Material 1.

## Data Availability

No datasets were generated or analysed during the current study.
