# Supplementary material for: Spotlight on mentoring undergraduate medical students using evidence-based medicine
Source: BMC Med Educ. 2026 May 25;26:1172. doi: 10.1186/s12909-026-09490-3 (PMC13377714; doi:10.1186/s12909-026-09490-3)
Supplement: Supplementary file 1 — Supplementary Material 1. [file 12909_2026_9490_MOESM1_ESM.pdf]

Appendix . **The full search string of this study** with this Boolean combination: “Evidence-based medicine" OR “EBM”) AND "undergraduate medical students" AND "clinical rotation”. Therefore, based on the results of the bibliometric analysis, a second detailed analysis of the retrieved publications was done using PRISMA-informed narrative synthesis.

Publications not retained are preceded by **X**.

1 - For the Scopus database, the following articles were found with this Boolean combination: "evidence-based medicine" OR “EBM”) AND "undergraduate medical students" AND "clinical rotation" (n =188 677 documents)

- Foguem, C., Manckoundia, P.

26430859600;6602832421;

Experience of medical students mentoring in an acute geriatric unit: Use of evidence-based medicine

(2019) European Journal of Internal Medicine, 60, pp. e23-e25. https://www.scopus.com/inward/record.uri?eid=2-s2.0-85060847376&doi=10.1016%2fj.ejim.2018.11.008&partnerID=40&md5=0b4b3f7dffa5f5b2261b3673f08b91ba. DOI: 10.1016/j.ejim.2018.11.008

- X - Nie, Y., Li, L., Duan, Y., Chen, P., Barraclough, B.H., Zhang, M., Li, J.

55509071800;57196177980;23484799300;55427529900;35551691100;57219086229;56085707000;

Patient safety education for undergraduate medical students: A systematic review (2011) BMC Medical Education, 11 (1), art. no. 33, . Cited 69 times. https://www.scopus.com/inward/record.uri?eid=2-s2.0-79958245217&doi=10.1186%2f1472-6920-11-33&partnerID=40&md5=d4518a3c334415b69efc2089256dbc5d. DOI: 10.1186/1472-6920-11-33 (Not selected because Review article)

2 - For the Sciences-direct, the following articles were found with this Boolean combination: "evidence-based medicine" OR “EBM”) AND "undergraduate medical students" AND "clinical rotation" 14 articles :

□ **Experience of medical students mentoring in an acute geriatric unit: Use of evidence-based medicine**. European Journal of Internal *Medicine*. February 2019. Clovis Foguem and Patrick Manckoundia

□ X Research article, Open access. **Science of Health Care Delivery: An Innovation in Undergraduate Medical Education to Meet Society's Needs.** Mayo Clinic Proceedings: Innovations, Quality & Outcomes. September 2017. Stephanie R. Starr, Neera Agrwal, C. Daniel Johnson (Not retained because the article does not specifically evaluate EBM)

□ X Research article, Open access. **Design, implementation and evaluation of a spiral module combining data science, digital health and evidence-based medicine in the undergraduate medical curriculum: A mixed methods study**. Clinical Medicine. May 2024. Nader Al-Shakarchi, Jaya Upadhyay, Amitava Banerjee. (Not retained because the article does not specifically evaluate EBM, but mainly focus on Digital health, data science and health informatics)

□ X Research article, Open access. **Prevalence of insomnia and related psychological factors with coping strategies among medical students in clinical years during the COVID-19 pandemic.** Saudi Journal of Biological Sciences. November 2021. Fahad Abdulaziz Alrashed, Kamran Sattar, Abdulrahman Mohammed Alsubiheen. Johnson (Not retained because the article does not evaluate EBM)

□ X Research article, Open access. **Medical education across three colleges of medicine: perspectives of medical students**. Heliyon. November 2022. Qasim A. El-Dwair, Intisar Mustafeh, Ayman G. Mustafa. (Not retained because the article aimed to explore and evaluate various components of the medical education process but does not evaluate EBM)

□ X Research article. **Integration of Community Health Teaching in the Undergraduate Medicine Curriculum at the University of Toronto.** American Journal of Preventive Medicine. October 2011. Ian L. Johnson, Fran E. Scott, Jay Rosenfield (Not retained because the article does not evaluate EBM)

□ X **Subject Index.** Journal of Surgical Education. December 2025. No authors available (Not retained because the article has no authors available neither abstract)

□ X Research article Open access.**The effectiveness of integrating making every contact count into an undergraduate medical curriculum**. PEC Innovation.15 December 2024. Robyn Fletcher, Alexander Hammant, Bharathy Kumaravel (Not retained because the article does not evaluate EBM)

□ X Correspondence **Medical research in war-torn Syria: medical students' perspective** The Lancet. 23–29 June 2018. Tareq Al Saadi, Fatima Abbas, Fares Alahdab (Not retained because the article does not evaluate EBM, among other)

□ X Research articl. **Status of Canadian undergraduate medical education in ophthalmology**. Canadian Journal of Ophthalmology October 2018; Mišo Gostimir. Rahul A. Sharma; Adil Bhatti (Not retained because the article does not evaluate EBM)

□ X Research article. **Fostering skills for evidence-based practice:** The student journal club Nurse Education in Practice. March 2006.Cathy J. Thompson (Not retained because the article does not evaluate EBM)

□ X Research articleOpen access.**Impact of Co-Designed Game Learning on Cultural Safety in Colombian Medical Education: Protocol for a Randomized Controlled Trial**; JMIR Research ProtocolsAugust 2020. Juan Pimentel, Anne Cockcroft, Neil Andersson (Not retained because the article does not evaluate EBM)

□ X Research articleOpen archive. **Development of a case-based integrated nutrition curriculum for medical students**. The American Journal of Clinical Nutrition. September 2000. Lisa A Hark, Gail Morrison (Not retained because the article does not evaluate EBM)

□ X Review article. **Library instruction and information literacy 2021**. Reference Services Review20 October 2022. Carolyn Caffrey, Hannah Lee, Katie Paris Kohn; (Not retained because the article does not evaluate EBM)

3 - The following 10 + 158 articles were selected from the Pubmed database, and only 19 finally retained.

Below is a shortlist of 40 articles from which we selected our final 19 articles, drawn from the 168 articles, abstracts, or article titles found on the PubMed search engine using the Boolean search term: ("evidence-based medicine" OR 'EBM') AND "undergraduate medical students" AND "clinical rotation".

[1] Teaching **evidence-based medicine**: Impact on **students**' literature use and inpatient **clinical** documentation.

Sastre EA, Denny JC, McCoy JA, McCoy AB, Spickard A 3rd.Med Teach. 2011;33(6):e306-12. doi: 10.3109/0142159X.2011.565827.PMID: 21609166.

[2] Experience of **medical students** mentoring in an acute geriatric unit: Use of **evidence-based medicine**.

Foguem C, Manckoundia P. Eur J Intern Med. 2019 Feb;60:e23-e25. doi: 10.1016/j.ejim.2018.11.008. Epub 2018 Nov 26.PMID: 30497765.

[3] Short course in **evidence-based medicine** improves knowledge and skills of **undergraduate medical students**: a before-and-after study.

Barghouti FF, Yassein NA, Jaber RM, Khader NJ, Al Shokhaibi S, Almohtaseb A, AbuRmaileh N.Teach Learn Med. 2013;25(3):191-4. doi: 10.1080/10401334.2013.797348.PMID: 23848323.

[4] X Integration of **evidence based medicine** into the **clinical** years of a **medical** curriculum.

Ferwana M, Alwan IA, Moamary MA, Magzoub ME, Tamim HM.J Family Community Med. 2012 May;19(2):136-40. doi: 10.4103/2230-8229.98307.PMID: 22870419 **Free PMC article.** (Study not retained because the number of participants included in the study is not specified and the study design (methodology) and results do not demonstrate a formal (comparative) evaluation of the participants or groups of participants).

[5] Two strategies to intensify **evidence-based medicine** education of **undergraduate students**: a randomised controlled trial.

Cheng HM, Guo FR, Hsu TF, Chuang SY, Yen HT, Lee FY, Yang YY, Chen TL, Lee WS, Chuang CL, Chen CH, Ho T.Ann Acad Med Singap. 2012 Jan;41(1):4-11.PMID: 22499474 **Free article.** Clinical Trial.

[6] **Evidence-based medicine** for **medical students**: introducing **EBM** in a primary **care rotation**.

Cayley WE Jr.WMJ. 2005 Apr;104(3):34-7.PMID: 15966630.

[7] Teaching and evaluating first and second year medical students' practice of evidence-based medicine.

Holloway R, Nesbit K, Bordley D, Noyes K. Med Educ. 2004 Aug;38(8):868-78. doi: 10.1111/j.1365-2929.2004.01817.x. PMID: 15271048.

[8] Evidence-based medicine (EBM) for undergraduate medical students.

Taheri H, Mirmohamadsadeghi M, Adibi I, Ashorion V, Sadeghizade A, Adibi P. Ann Acad Med Singap. 2008 Sep;37(9):764-8. PMID: 18989493.

[9] Evaluation of a longitudinal **medical** school **evidence-based medicine** curriculum: a pilot study.

West CP, McDonald FS.J Gen Intern Med. 2008 Jul;23(7):1057-9. doi: 10.1007/s11606-008-0625-x.PMID: 18612744 **Free PMC article.**

[10] X Teaching critical appraisal: a pilot randomized controlled outcomes trial in **undergraduate** osteopathic **medical** education.

Krueger PM.J Am Osteopath Assoc. 2006 Nov;106(11):658-62.PMID: 17192453 Clinical Trial. (Not retained because the article doesn't focus on medical students but on osteopathy students.).

[11] Preparing for Pediatrics: Experiential Learning Helps **Medical Students** Prepare for Their **Clinical** Placement.

Sullivan C, Condron C, Mulhall C, Almulla M, Kelly M, O'Leary D, Eppich W.Front Pediatr. 2022 Mar 4;10:834825. doi: 10.3389/fped.2022.834825. eCollection 2022.PMID: 35311060.

[12] X A novel approach to incorporating **evidence-based medicine** into an emergency **medicine** clerkship.

Snashall J, Fair M, Scott J.Acad Emerg Med. 2013 Mar;20(3):295-9. doi: 10.1111/acem.12089.PMID: 23517262 **Free article.** (Article not retained and included, despite the "new and interesting approach described for integrating EBM during Emergency Medicine training courses" proposed, due to the absence of figures or comparison of the groups of students included with the statistical analyses carried out in this article. Nevertheless, elements of this article have been used in the Discussion section of our paper).

[13] Effectiveness of Modalities to Teach **Evidence Based Medicine** to Pediatric Clerkship **Students**: A Randomized Controlled Trial.

Hadvani T, Dutta A, Choy E, Kumar S, Molleda C, Parikh V, Lopez MA, Lui K, Ban K, Wallace SS.Acad Pediatr. 2021 Mar;21(2):375-383. doi: 10.1016/j.acap.2020.09.012. Epub 2020 Sep 30.PMID: 33010470 **Free PMC article**.

[14] Çakmakkaya ÖS. Formal evidence-based medicine instruction in Turkish undergraduate medical education: an initial evaluation. BMC Med Educ. 2021 Aug 19;21(1):437. doi: 10.1186/s12909-021-02876-5. PMID: 34407804 Free PMC article.

[15] Kumaravel B, Jenkins H, Chepkin S, Kirisnathas S, Hearn J, Stocker CJ, Petersen S. A prospective study evaluating the integration of a multifaceted evidence-based medicine curriculum into early years in an undergraduate medical school. BMC Med Educ. 2020 Aug 24;20(1):278. doi: 10.1186/s12909-020-02140-2. PMID: 32838775 Free PMC article.

[16] Liabsuetrakul T, Sutharasaj T, Tangtrakulwanich B, Uakritdathikarn T, Pornsawat P. Longitudinal analysis of integrating evidence-based medicine into a medical student curriculum. Fam Med. 2009 Sep;41(8):585-8.PMID: 19724944.

[17] X Ilic D, Nordin RB, Glasziou P, Tilson JK, Villanueva E. A randomised controlled trial of a blended learning education intervention for teaching evidence-based medicine. BMC Med Educ. 2015 Mar 10;15:39. doi: 10.1186/s12909-015-0321 - 6.PMID: 25884717 . (Not retained because the article did not evaluate EBM itself but compared the effectiveness of two learning methods applied to EBM blended learning (BL) versus didactic learning (DL) approach of teaching EBM to medical students).

[18] Ghojazadeh M, Hajebrahimi S, Azami-Aghdash S, Pournaghi Azar F, Keshavarz M, Naghavi-Behzad M, Hazrati H. Medical students' attitudes on and experiences with evidence-based medicine: a qualitative study. J Eval Clin Pract. 2014 Dec;20(6):779-85. doi: 10.1111/jep.12191. Epub 2014 Jul 16.PMID: 25039542.

[19] X Hosny S, Ghaly MS. Teaching evidence-based medicine using a problem-oriented approach. Med Teach. 2014 Apr;36 Suppl 1:S62-8. Doi/ 10.3109/0142159X.2014.886007.PMID: 24617787 (Not retained because the article did not evaluate EBM only but EBM has been integrated here into problem based learning (PBL) sessions, for sixth-year or final year medical students. In this study, not only students were assessing, but also and tutor).

[20] Srinivasan M, Weiner M, Breitfeld PP, Brahmi F, Dickerson KL, Weiner G. Early introduction of an evidence-based medicine course to preclinical medical students.J Gen Intern Med. 2002 Jan;17(1):58-65. doi: 10.1046/j.1525-1497.2002.10121.x.PMID: 11903776 **Free PMC article**.

[21] X Ilic D, Diug B. The impact of clinical maturity on competency in evidence-based medicine: a mixed-methods study. Postgrad Med J. 2016 Sep;92(1091):506-9. doi: 10.1136/postgradmedj-2015-133487. Epub 2016 Feb 11.PMID: 26869718. (Not retained because in this study, undergraduate and graduate-entry medical trainees entering their first year of training were included, and the two groups were compared, study not focuses only on undergraduate medical students).

[22] Elçin M, Turan S, Odabaşı O, Sayek I. Development and evaluation of the evidence-based medicine program in surgery: a spiral approach. Med Educ Online. 2014 Apr 25;19:24269. doi: 10.3402/meo.v19.24269. eCollection 2014.PMID: 24767706 .

[23] Aronoff SC, Evans B, Fleece D, Lyons P, Kaplan L, Rojas R. Integrating evidence based medicine into undergraduate medical education: combining online instruction with clinical clerkships.Teach Learn Med. 2010 Jul;22(3):219-23. doi: 10.1080/10401334.2010.488460.PMID: 20563945.

[24] Liabsuetrakul T, Suntharasaj T, Sangsupawanich P, Kongkamol C, Pornsawat P. Implementation of evidence-based medicine in a health promotion teaching block for Thai medical students. Glob Health Promot. 2017 Dec;24(4):62-68. doi: 10.1177/1757975915626871. Epub 2016 May 6.PMID: 27154911.

[25] Lai NM, Nalliah S. Information-seeking practices of senior medical students: the impact of an evidence-based medicine training programme. Educ Health (Abingdon). 2010 Apr;23(1):151. Epub 2010 Apr 9.PMID: 20589599.

[26] X Somers AR, Warburton SW, Moolten S. Teaching geriatric care: report on an experimental second-year elective. J Fam Pract. 1978 Mar;6(3):573-8.PMID: 632769 (Not retained because the article did not evaluate EBM).

[27] X Kataoka Y, Maeno T, Inaba T, Ninn S, Suzuki M, Maeno T. A qualitative study of factors promoting EBM learning among medical students in Japan. Int J Med Educ. 2022 Aug 26;13:215-220. doi: 10.5116/ijme.62eb.7c19.PMID: 36036207 **Free PMC article..** (Not retained because the participants were physicians working at universities, teaching hospitals, or clinics who teach EBM to medical students, but not undergraduate medical students).

[28] X Rees E, Sinha Y, Chitnis A, Archer J, Fotheringham V, Renwick S. Peer-teaching of evidence-based medicine. Clin Teach. 2014 Jul;11(4):259-63. doi: 10.1111/tct.12144.PMID: 24917093 (Not retained because EBM workshops were organized and delivered by fourth-year medical students, having first received training from NICE to

become NICE student champions; and it is not specify whether the participants were exclusively undergraduate medical students)

[29] X Snashall J, Fair M, Scott J. A novel approach to incorporating evidence-based medicine into an emergency medicine clerkship. .Acad Emerg Med. 2013 Mar;20(3):295-9. doi: 10.1111/acem.12089.PMID: 23517262 **Free article.** (Not retained even the authors have developed a novel fourth-year EM clerkship curriculum that integrates EBM through the use of a highly interactive, faculty-led, small group-learning environment that encourages students to develop the necessary skills, but the actual comparative evaluation of EBM among undergraduate medical students was not carried out).

[30] X Buljan I, Jerončić A, Malički M, Marušić M, Marušić A. How to choose an evidence-based medicine knowledge test for medical students? Comparison of three knowledge measures. BMC Med Educ. 2018 Dec 4;18(1):290. doi: 10.1186/s12909-018-1391-z.PMID: 30514288 **Free PMC article.** (Not retained because the participants were the first 3 years of a 6-year medical graduate program).

[31] X Varan AK, Winkel K, McKenzie R. Your Quest Begins Now! EBMQuest, a Digital Interactive Fiction Module for Medical Student Engagement in Evidence-Based Practice. Stud Health Technol Inform. 2022 Jun 6;290:917-918. doi: 10.3233/SHTI220213.PMID: 35673152. (Not retained because it an online interactive fiction module "EBMQuest and and it is not specify whether the participants were exclusively undergraduate medical students).

[32] X Forjuoh SN, Rascoe TG, Symm B, Edwards JC. Teaching medical students complementary and alternative medicine using evidence-based principles. J Altern Complement Med. 2003 Jun;9(3):429-39. doi: 10.1089/107555303765551651.PMID: 12816631. (Not retained because the study did not only focus on EBM, but evaluated the effectiveness of teaching CAM using EBM principles and assessed changes in student perceived knowledge, attitudes, and skills following a new curriculum on CAM).

[33] X Marušić A, Malički M, Sambunjak D, Jerončić A, Marušić M. Teaching science throughout the six-year medical curriculum: two-year experience from the University of Split

School of Medicine, Split, Croatia.. Acta Med Acad. 2014;43(1):50-62. doi: 10.5644/ama2006-124.100.PMID: 24893639 **Free article.** (Not retained because the aim of the study was to present the introduction of a mandatory, vertically integrated course in research methodology into medical curriculum. Even if the third year, 25 hours was devoted to mastering concepts and basic skills of Evidence based medicine (EBM), the evaluation of EBM is not the primary objective of the study).

[34] X Lucchetti AL, da S Ezequiel O, Moreira-Almeida A, Lucchetti G . Measuring medical students' attitudes and knowledge about geriatrics and gerontology in Brazilian medical students: A comparison of instruments. Australas J Ageing. 2018 Jun;37(2):E74-E77. doi: 10.1111/ajag.12532. Epub 2018 Apr 14.PMID: 29655239. (Not retained because, the objective of this study was to compare instruments available for evaluating attitudes and knowledge about geriatrics and gerontology, but to evaluate EBM).

[35] X Markert RJ. EBM and biostatistics courses. Acad Med. 1998 Oct;73(10):1028-9. doi: 10.1097/00001888-199810000-00002.PMID: 9795612 No abstract available. (Not retained because this article (editorial or letter) does not assess EBM among undergraduate medical students).

[36] X Duque G. Community implementation of evidence-based interventions in geriatric medicine: Time to translate research into practice. Arch Gerontol Geriatr. 2023 Mar;106:104914. doi: 10.1016/j.archger.2022.104914. Epub 2022 Dec 26.PMID: 36592556 No abstract available. (Not retained because this article does not assess EBM among undergraduate medical students).

[37] X Lim WS, Ding YY. Evidence-balanced medicine: "real" evidence-based medicine in the elderly. Ann Acad Med Singap. 2015 Jan;44(1):1-5.PMID: 25703490 **Free article.** No abstract available. (Not retained because this article does not assess EBM among undergraduate medical students).

[38] X Atwa H, Abdelaziz A. Evidence-based medicine (EBM) for undergraduate medical students: A six-step, integrative approach. Med Teach. 2017 Apr;39(sup1):S27-S32. doi: 10.1080/0142159X.2016.1254750. Epub 2017 Jan 20.PMID: 28103721 (Not retained, although the main of the study was to design and pilot a basic course on EBM for undergraduate medical students in order to raise the awareness of the Saudi medical education community about this discipline; because the course was integrated longitudinally into Integrated Multisystem Module of Year 4, but not the specific EBM assessment. Students and faculty perceptions were evaluated for program evaluation purposes).

[39] X Vidyarthi A, Lek N, Chan K, Kamei R. Experiences with a clinical reasoning and evidence-based medicine course. Clin Teach. 2016 Feb;13(1):52-7. doi: 10.1111/tct.12362. Epub 2015 May 27.PMID: 26013658 (Not retained, because participants were final-year medical students and the purpose of the study was to describe and characterise student experiences with a clinical reasoning and EBM curriculum).

[40] X Finkel ML, Brown HA, Gerber LM, Supino PG. Teaching evidence-based medicine to medical students. Med Teach. 2003 Mar;25(2):202-4. doi: 10.1080/0142159031000092634.PMID: 12745533 (Not retained, because this report reviews the process of implementing a four-week comprehensive course in the concepts and techniques of EBM focused on methods, study design and statistical analysis in assigned articles as well as on instruction in database-searching techniques; but it's not a formal evaluation of EBM).
